# Expansion, isolation and first characterization of bovine Th17 lymphocytes

**DOI:** 10.1038/s41598-019-52562-2

**Published:** 2019-11-06

**Authors:** Patricia Cunha, Yves Le Vern, Christophe Gitton, Pierre Germon, Gilles Foucras, Pascal Rainard

**Affiliations:** 1 ISP, INRA, Université de Tours, UMR1282 Nouzilly, France; 2 IHAP, INRA, ENVT, Université de Toulouse, Toulouse, France

**Keywords:** Immune cell isolation, T-helper 17 cells

## Abstract

Interleukin 17A-producing T helper cells (Th17) are CD4+ T cells that are crucial to immunity to extracellular bacteria. The roles of these cells in the bovine species are poorly defined, because the characterization of bovine Th17 cells lags behind for want of straightforward cultivation and isolation procedures. We have developed procedures to differentiate, expand, and isolate bovine Th17 cells from circulating CD4+ T cells of adult cows. Using polyclonal stimulation with antibodies to CD3 and CD28, we expanded IL-17A-positive CD4+ T cells in a serum-free cell culture medium supplemented with TGF-β1, IL-6 and IL-2. Populations of CD4+ T cells producing IL-17A or IFN-γ or both cytokines were obtained. Isolation of IL-17A-secreting CD4+ T cells was performed by labelling surface IL-17A, followed by flow cytometry cell sorting. The sorted Th17 cells were restimulated and could be expanded for several weeks. These cells were further characterized by cytokine profiling at transcriptomic and protein levels. They produced high amounts of IL-17A and IL-17F, and moderate amounts of IL-22 and IFN-γ. The techniques developed will be useful to characterize the phenotypic and functional properties of bovine Th17 cells.

## Introduction

IL-17A-producing CD4+ helper T cells, defined as Th17 cells, have been identified as a distinct helper T cell lineage^1^, on the basis of the production of IL-17A, a few associated phenotypic markers such as the expression of the chemokine receptor CCR6 and the cytokine IL-23 receptor, and the expression of the lineage-specifying transcription factor retinoic acid-related orphan receptor RORγt^2^. The Th17 cell lineage is involved in the defence against a variety of pathogens and has been linked to the pathogenesis of several inflammatory and autoimmune diseases^3^. For these reasons, Th17 cells are under intense investigation. They are among the effector cells of the type 3 cell-mediated immunity, comprising lymphocytes expressing retinoic acid orphan receptor *RORC* coding RORγt, and producing IL-17A, IL-17F alone or in combination with IL-22 as signature cytokines^4^. Th17 cells are particularly adapted to the protection of epithelial sites against extracellular bacteria and fungi, mainly through the activity of their effector cytokines on cells that express the IL-17 receptor^5^. Th17 cells and IL-17A have been shown to play an important role in host defence against Gram-positive or negative bacteria and fungi in the lungs, intestine and mammary gland^6–9^. There are reasons to think that IL-17-producing cells play a role in the defence of the mammary gland of dairy ruminants against bacterial infections. Bovine mammary epithelial cells are responsive to IL-17A and IL-17F, and these cytokines are induced in the udder tissues of mammary glands infected by *Escherichia coli* or in milk of cows or goats infected by *E. coli* or *Staphylococcus aureus*^10–12^. The immune response to other bovine infections can be influenced by IL-17A and Th17 cells, such as bovine tuberculosis or paratuberculosis^13–15^.

Most of the information on Th17 cells has been generated in mouse models and studies on human T cells. Comparably little is known about bovine Th17 cells, for want of established cultivation and isolation procedures. Currently, bovine CD4+ Th17 cells are defined on the sole basis of their ability to produce IL-17A, evidenced by intracellular staining with antibodies to IL-17A^16–19^. This procedure entails fixation and permeabilization of the cells, and consequently prevents experimenters from doing functional studies on bovine Th17 cells. This is why we set out to develop a procedure to isolate these cells and to maintain and expand them in culture so that functional studies can be done. We adapted to bovine cells methods that have been developed for the isolation of live mouse or human Th cells with cytometric cytokine secretion assays^20–22^. Cultivation of bovine CD4+ T cells under polarizing condition followed by flow cytometry cell sorting using cell surface IL-17A detection yielded IL-17A+ CD4+ T cells that could be grown *in vitro* for several weeks. The validation of straightforward procedures for cultivation and expansion of viable bovine Th17 cells, making use of commercially available reagents and serum free medium, will make it possible to characterize the generation, regulation and functions of this cellular lineage and its comprising cellular subsets. The acquired new knowledge will be useful for developing procedures to study and modulate the type 3 arm of the adaptive T cell response in the bovine species.

## Materials and Methods

### Ethics statement

The procedure involving animals (blood sampling) received approval from the Ethics Committee of Val de Loire (agreement no. 4809 INRA). Blood sampling was performed by authorized staff members in accordance with the relevant standard operating procedures approved by the above-mentioned Ethics Committee. All animals, of the permanent dairy herd of the INRA experimental Unit UE-PAO (Nouzilly, agreement n° F37-175-2) were handled in strict accordance with good clinical practices.

### Isolation, culture and surface marker labelling of CD4+ T cells

Three healthy cows were used as blood donors for the purification of PBMC. Blood samples were collected in 10-mL tubes coated with EDTA (Venosafe™, Terumo® Europe). PBMC were prepared as described^16^, by centrifugation to obtain the buffy coat before transfer onto a Percoll cushion, centrifugation and collection of the white blood cell layer. CD4+ lymphocytes were then purified by positive selection using MACS® beads according to the manufacturer’s instructions (Miltenyi Biotech, Bergish Gladbach, Germany). Briefly, PBMC were incubated with a mouse anti-bovine CD4 (Bio-Rad AbD Serotec, clone CC30) for 20 min. After washing, cells were labelled with anti-mouse IgG MACS microbeads in MiniMACS buffer (PBS, 2 mM EDTA, 0.5% bovine serum albumin) for 20 min under mild agitation. CD4+ cells were isolated by passage over a MACS® (MS) separation column mounted on an OctoMACS® separator. Cells were washed and resuspended in the serum free X-VIVO™ 15 Hematopoietic cell medium (LONZA) supplemented with 2 mM L-glutamine, 10 mM HEPES, penicillin-streptomycin and fungizone. The purity of the CD4+ population, as assessed by fluorescence flow cytometry, was consistently over 91%.

In preliminary experiments, we compared several culture media with or without foetal calf serum (FCS): RPMI 1640 plus 10% FCS, Iscove’s modified Dulbecco’s medium (IMDM) supplemented with 10% KnockOut™ Serum Replacement (Gibco), and TexMACS™ medium (Miltenyi Biotech).

Cell surface phenotyping was carried out by using mouse monoclonal antibodies to CD markers (CD4, CD45RO, CD62L) and secondary antibodies listed in SupplementaryTable S1. Staining was carried out in the wells of round-bottomed microtitre plates (Falcon™) in a volume of 100 µL for 10^6^ cells for 20 min in the dark at 4 °C, followed by two washes, with appropriate isotype-matched controls.

Plate-bound T cell receptor stimulation was performed by distributing 10^6^ CD4+ T cells per well of flat-bottomed 24-well microplates (Falcon™) coated with 5 µg/mL anti-CD3 monoclonal antibody (clone MMIA, Kingfisher Biotech) in a Th17 polarizing culture medium made up of X-VIVO™ 15 supplemented with 2 µg/mL anti-CD28 (clone CC20, Bio-Rad AbD Serotec), 40 ng/mL recombinant human IL-6 (Peprotech) and 2 ng/mL recombinant human TGF-β1 (Peprotech). The activity of human recombinant IL-6 on bovine cells has been documented^23^. Cells were cultured for 3 days at 38.5 °C (physiological body temperature of cows) under 5% CO_2_ and adjusted to 10^6^/mL with medium (without anti-CD28) renewal and addition of 10 ng/mL of recombinant human IL-2 (Peprotech), which has been shown to be active on bovine lymphocytes^24^. This operation was renewed every third day. On occasion, CD45RO surface expression was measured by flow cytometry by labelling with a mouse monoclonal (ILA116A, Kingfisher Biotech) followed by PerCP-conjugated goat anti-mouse IgG3 (Jackson Immunoresearch).

**Figure 1:**
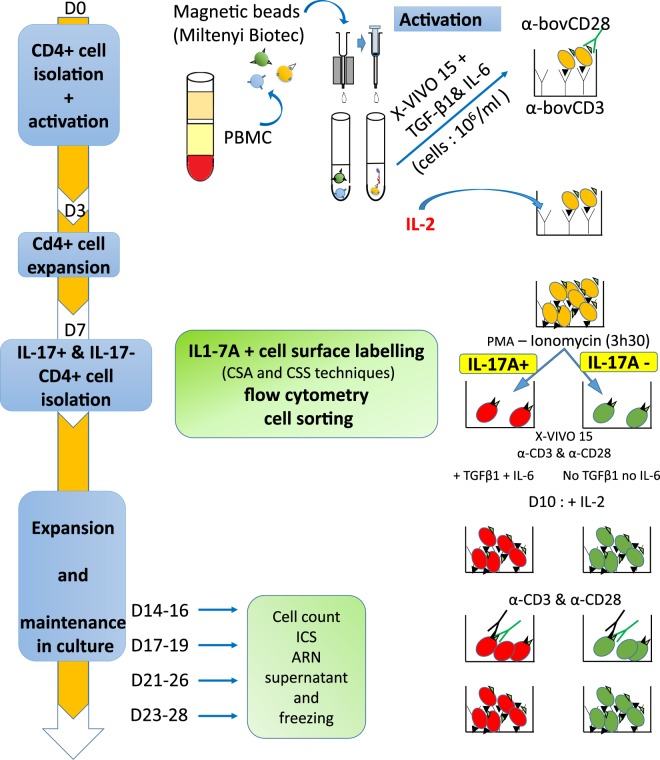
Schematic representation of the procedure used for the isolation and expansion of Th17 cells. CD4+ cells were isolated from PBMC by magnetic sorting, expanded in a culture medium without serum in the presence of polarizing cytokines and TCR-stimulation, activated, labelled for surface IL-17A and sorted by flow cytometry. The sorted cells were expanded and maintained in culture for several weeks. The indicated timeline is kept in all the figures to make it clear at which step cells were assayed. Concentrations of cytokines were 10 ng/mL (IL-2), 40 ng/mL (IL-6) and 2 ng/mL (TGF-β1). D: day; CSA: cytokine secretion assay; CSS: cytokine surface staining. α-: antibody anti-.

The timeline of the chain of interventions leading from PBMC to expanded Th17 cells is presented in Fig. 1.

### Flow cytometry analysis of intracellular cytokines

**Figure 2:**
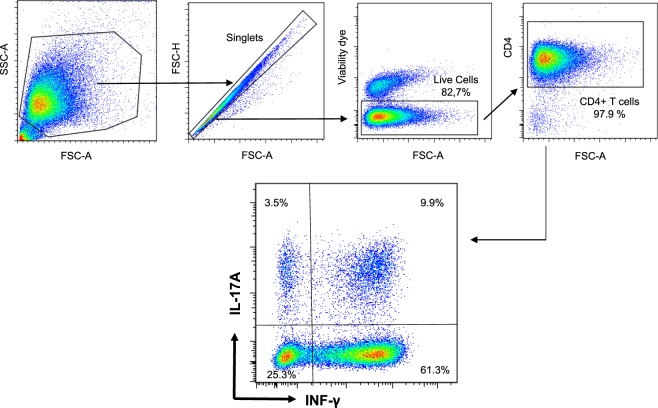
Identification of IL-17A and IFN-γ producing bovine lymphocytes. PBMC were stimulated with PMA/ionomycin, cytokine secretion blocked with Brefeldin A before flow cytometry analysis. Debris were excluded by gating according to FSC/SSC, and after gating on singlet cells, dead cells were excluded by gating on live cells. The CD4+ cells were gated by taking into account the isotype control, and the production of IL-17A and IFN-γ was measured by intracellular labelling with specific antibodies. Results shown are from a representative experiment. FSC: forward scatter: SSC: side scatter.

To induce cytokine production, CD4+ T cells were stimulated for 5 h with 50 ng/mL phorbol 12-myristate 1-acetate (PMA) and 500 ng/mL ionomycin (Sigma) in the presence of Brefeldin A 10 µg/ml (Sigma). Cells were washed with Dulbecco’s PBS, distributed in round-bottomed 96 plates (10^6^ cells/well) and stained with a live/dead cell stain kit (Fixable Viability Dye eFluor® 450, eBioscience) then surface stained with fluorescent anti-CD4 (clone CC8 conjugated to A647, Bio-Rad) in FACS buffer (DPBS supplemented with 2 mM EDTA and 2% goat serum). Cells were permeabilized using the Cytofix/Cytoperm kit (BD Pharmingen). Intracellular cytokines were labelled with anti-human IL-17A-PE-CY7 (DEC17, eBioscience) and anti-bovine IFN-γ-A488 (clone CC302, Bio-Rad) in Perm/Wash™ buffer (BD Bioscience) in a 50 µL volume for 10^6^ cells for 30 min at room temperature in the dark (see Supplementary Table S1). After washing and resuspension in FACS buffer, the cells were examined by flow cytometry using a BD LSR Fortessa cytometer and data were analyzed with the FlowJo software (Tree Star, Ashland, OR, USA). Gates were set according to appropriate isotype/control staining. A first gate was set up on the forward scatter (FSC)/side scatter (SCC) plot to remove debris, then single cells were gated, followed by a gate on live cells (Fig. 2). A minimum of 50,000 events were acquired.

### IL-17 cytokine secretion assay (CSA)

The cytokine secretion assay makes use of a capture complex that assembles an antibody to the leucocyte surface antigen CD45 and an antibody to IL-17A^20^. The antibodies are biotinylated and joined by a streptavidin molecule. Monoclonal antibodies to the N-terminal and C-terminal sequences of bovine IL-17A were developed (ProteoGenix SAS, Schiltigheim, France). We selected the hybridoma subclones most reactive with recombinant bovine IL-17A (clone 2.1.1, peptide GVIIPQSPGC, N-ter and clone 24.2.4, peptide CVTPIVAHLA, C-ter). These monoclonal antibodies do not react with recombinant bovine IL-17F in Western blot or indirect ELISA. To assemble the complex, biotinylated mouse anti-sheep CD45 (400 ng; clone 1.11.32, Bio-Rad) was mixed with 5 µg biotinylated mouse monoclonal antibody to bovine IL-17A (anti-N terminal peptide). After thorough vortexing, 5 µg of streptavidin (Sigma) was added along with DPBS supplemented with 2 mM EDTA and 2% goat serum (total volume 27 µL). The complex was incubated for 10 min at room temperature and vortexed again before use. The obtained complex was used to treat 10^6^ CD4+ T cells.

Before capture, CD4+ T cells polarized for 7 to 9 days, suspended in X-VIVO™ 15 medium (10^6^/mL), were stimulated with 50 ng/mL PMA and 500 ng/mL ionomycin for 3.5 h at 38.5 °C. A secretion negative control was prepared in parallel by adding 10 µg/mL brefeldin A. For surface labelling with the capture complex, the cells were washed with cold FACS buffer, pelleted by centrifugation, and resuspended in 75 µL FACS buffer before addition of 25 µL capture complex. Cells were incubated for 15 min on ice, allowing the capture complex to bind to CD45 positive cells. Cells were then diluted with 10 mL X-VIVO™ 15 medium and incubated for 1.5 h under mild agitation at 38.5 C. The high dilution and agitation are to reduce the risk of cross-labeling of bystander non-secreting cells. At the end of the secretion step, 10 mL of cold FACS buffer was added and the cells were centrifuged (400 *g*, 7 min at 4 °C). Cells were incubated with 5 µg/mL affinity-purified rabbit antibodies to bovine IL-17A (Kingfisher Biotech) for 20 min at 4 °C followed by labelling with 2.5 µg/mL donkey anti-rabbit IgG-PE (Jackson Immunoresearch) for 20 min at 4 °C, and then were stained with the live/dead cell stain kit. Between each step, cells were washed twice with cold FACS buffer. The combination of antibodies used is presented in Supplementary Table S2.

### Direct labelling of surface IL-17A for cytokine surface staining (CSS)

As an alternative to the complex capture assay (CSA), we performed the direct labelling of surface IL-17A with specific antibodies, as described for human Th17 cells^21^. Cells were prepared and stimulated as for the CSA. After washing at the end of the 3.5 h stimulation, cells were incubated with 5 µg/mL affinity-purified rabbit antibodies to bovine IL-17A (Kingfisher Biotech) for 20 min at 4 °C, followed by labelling with 2.5 µg/mL donkey anti-rabbit IgG-PE (Jackson Immunoresearch) for 20 min at 4 °C, then stained for viability with the live/dead cell stain kit.

### Sorting of IL-17A+ cells

IL-17-labeled cells were sorted using the cell sorter MoFlo Astrios EQ (Beckman Coulter, Brea, CA, USA). Cells were dispersed (2 × 10^6^/mL) in PBS and sorting was performed at 40 psi using a 90 µm nozzle. IL-17A− and IL-17A+ cells were collected in two Eppendorf tubes containing 500 µL X-VIVO™ 15 medium.

### Long-term culture of sorted T cells

Sorted T cells were pelleted and resuspended in 500 µL X-VIVO™ 15 medium in 48-well culture plate. The medium was supplemented with soluble anti-CD3 (2.5 µg/ml) and anti-CD28 (2 µg/ml) antibody for IL-17- and IL-17+ cells, the latter receiving also the polarizing cytokines IL-6 and TGF-β1 at the same concentrations as before sorting. Recombinant IL-2 was added after 3 days of TCR stimulation at 38.5 °C for both cell types. The media were half-renewed every third day, and cells were diluted at 10^6^/mL when necessary.

When the cells were well grown, at least 10^6^ cells were harvested, washed in DPBS and resuspended in 1 ml of cold freezing media (FBS + 10% DMSO). The cryovials were put in a freezing container and immediately stored at −80 °C. After 24 h, the cryovials were transferred into liquid nitrogen for long term storage. For thawing, the cryovials were placed in a 37 °C water bath until only about 3/4 defrosted. Then, the content of the vial was delicately transferred in 15 ml of pre-warmed X-VIVO™ 15 medium and centrifuged at 400 *g* for 8 min. The cells were then resuspended at 10^6^/ml in X-VIVO™ 15 medium + 40 ng/mL IL-6 + 2 ng/mL TGF-β1 and 10 ng/ml IL-2. Cells were cultured at 38.5 °C under 5% CO_2_ and adjusted to 10^6^/mL with the same culture media. At day 13 after thawing, a new T cell receptor (TCR) stimulation (CD3/CD28 antibodies) was performed.

### ELISA measurement of cytokine production

Cytokine production by CD4+ T cells was measured by Enzyme-linked immunosorbent assays (ELISAs). A commercially available kit was used for IFN-γ (Mabtech AB, Nacka Strand, Sweden). In-house ELISAs were used for bovine IL-17A, IL-17F and IL-22 as described^25^ and detailed in Supplementary Table S3.

### Reverse transcription and real-time quantitative PCR

After purification on MACS columns from PBMC, CD4+ cells were stimulated with anti-CD3 and anti-CD28 antibodies and cultured for 6 days in X-VIVO™ 15 medium. Cultures were carried out (with addition of recombinant IL-2 at day 3 with or without the polarizing cocktail of cytokines (TGF-β1 and IL-6). The cells cultured under non-polarizing condition served as reference for the RT-qPCR analysis. In parallel, 0.5 to 2.10^6^ IL-17A+ and IL-17A− cells were cultured under polarizing condition and harvested at different times. All cells were lysed in RA1 + 1% β-mercaptoethanol buffer (Macherey-Nagel, Düren, Germany) and stored at −80 °C. Total RNA was extracted by using the NucleoSpin RNA extraction kit (Macherey-Nagel), and the residual genomic DNA was removed by using DNase digestion with RNase-free DNase (Macherey-Nagel). The total RNA quantity was assessed by using a NanoDrop spectrophotometer (NanoDrop Technologies, Wilmington, DE). RNA integrity was analyzed using the Agilent Bioanalyzer System (Agilent technologies, Inc., Santa Clara, CA, USA). RNA samples RIN (RNA Integrity Number) was between 9.1 to 10. Total RNA (100 ng) was then reverse transcribed to cDNA using 5x iScript reverse transcription supermix (BIORAD) according to manufacturer’s instructions. cDNA samples were stored at −20 °C until use.

Primers used in this study are listed in Supplementary Table S4. PCR-primer pairs were designed from mRNA sequences of the studied genes provided by the National Center for Biotechnology Information (NCBI) gene database using PrimerBlast. Optimal Tm at 60 °C and exon junction span were selected. Primer pair oligos were ordered at 100 µM concentration with purification SePOP desalting (Eurogentec, Kaneka Corporation, Osaka, Japan).

Before using Fluidigm technology, each assay was validated with conventional RT-qPCR in a LightCycler 480 instrument. Four µl of tenfold diluted cDNA were added to a mix of iTaq universal SYBRGreen supermix (2X) (BIORAD) and 0.25 µM of each primer in a total volume of 10 µl. Thermal protocol was 95 °C for 5 min followed by 40 cycles of 95 °C for 10 sec, 60 °C for 30 sec and acquisition of a melting curve at the end of the run. Specificity of primer pairs was checked via melting curve analysis. No Reverse transcriptase and no template controls were analysed. PCR efficiencies (87 to 100%) were determined with a 5-point dilution series of PBMC cDNA. For the Fluidigm PCR, tenfold diluted cDNA was preamplified according to Fluidigm’s protocol (quick reference PN 100-5875 B1) and preamplified cDNA were diluted fivefold with Tris-EDTA buffer. Gene expression levels were measured on 48 × 48 GE Dynamic Array IFC using the Fluidigm BioMark^TM^ HD System. Fold changes were calculated by the ∆∆Ct method using “Fluidigm Real-Time PCR Analysis” software. Reference genes used were ACTB, PPIA and GAPDH^26^. For each batch of cells, the reference sample was the CD4+ cells after 6 days of culture without polarizing cytokines but with an initial TCR stimulation and a partial (half) renewal of medium with addition of IL-2 on the 3^rd^ day. Heatmaps were generated using the pheatmap package (v1.0.10) in RStudio (1.1).

## Results

### Culture conditions for expansion of Th17 cells

As the proportion of CD4+ IL-17A+ cells circulating in bovine blood is low^18^, our first goal was to increase this proportion to get IL-17A-positive CD4+ T cells in numbers sufficient for cell sorting. We started from blood T cells, likely to comprise a mixture of naïve and memory cells. The proportion of CD45RO+ cells was about 30% of CD4+ cells in the blood of the three donor cows. Purified CD4+ T cells were activated with immobilized anti-CD3 and soluble CD28 antibodies in RPMI medium supplemented with 10% FCS and the Th17 polarizing cytokines TGF-β and IL-6. At this stage, we sought to establish culture conditions suitable for the expansion of Th17 cells and for production of IL-17A.

**Figure 3:**
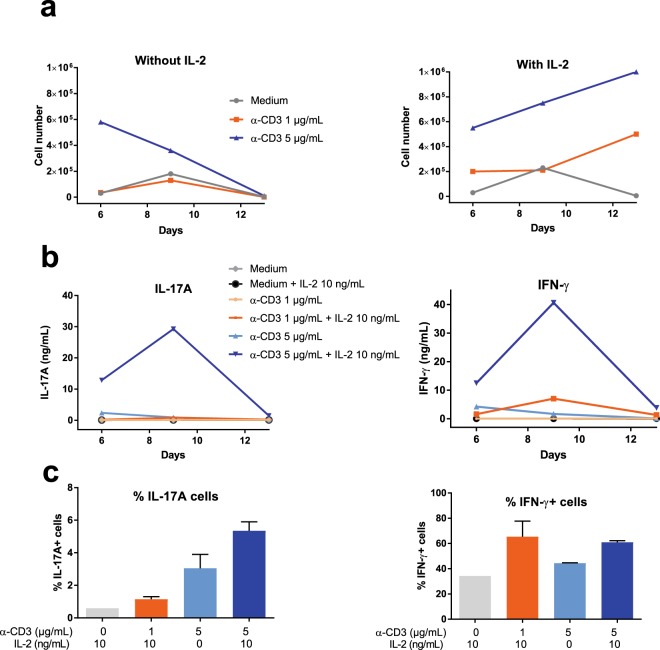
Culture conditions for expansion of Th17 cells. (**a**) Number of cells after 6, 9 and 13 days of culture as a function of the concentration (µg/mL) of the coating antibodies to CD3 with or without 10 ng/ml recombinant human IL-2. Results are from a representative experiment. (**b**) Concentrations of IL-17A and IFN-γ under these culture conditions. (**c**) Proportion of cells producing IL-17A or IFN-γ (ICS) at day 6 of culture. Results are means from the cells of two cows. Percentages of IL-17A positive cells differed as a function of treatment (p = 0.034, one-way ANOVA), but not percentages of IFN-γ positive cells (p = 0.24, one-way ANOVA). Results with α-CD3 1 µg/mL are not shown because too few cells were available for staining.

We investigated the effect of different amounts of anti-CD3 antibody and of a commonly used concentration of IL-2 (10 ng/mL) on the growth of IL-17A+ CD4+ T cells, as it has been reported that the proliferation of Th17 cells is hampered by high TCR stimulation or high concentrations of IL-2^27,28^. Purified CD4+ cells were distributed in wells coated with either 1 or 5 µg/mL anti-CD3 after resuspension in the polarizing medium and a constant concentration of soluble anti-CD28. After 3 days of culture the medium was renewed and IL-2 added (10 ng/ml) or not. At days 6, 9 and 13 the cells were enumerated, IL-17A and IFN-γ concentrations in culture supernatants were measured (ELISA) and the proportion of IL-17A and IFN-γ-positive cells established by intracellular staining (ICS). The highest anti-CD3 amount tested allowed the cells to grow more efficiently, and the presence of IL-2 was required for sustained growth of the cells beyond day 6 (Fig. 3a). Likewise, the secretion of IL-17A and IFN-γ was also at its best at day 9 with the highest anti-CD3 stimulus in the presence of IL-2 (Fig. 3b). The proportion of IL-17A+ cells augmented with increasing anti-CD3 amount, whereas IL-2 tended to increase the proportion of cells producing IL-17A or IFN-γ (Fig. 3c). It can also be seen that the best condition for IL-17 production (5 µg/mL anti-CD3 with 10 ng/mL IL-2) was also the best condition for the production of IFN-γ (Fig. 3b), although without increase in the proportion of cells positive for IFN-γ by ICS (Fig. 3c).

**Figure 4:**
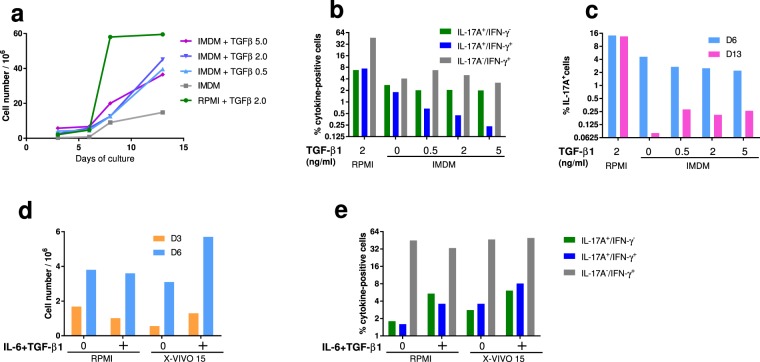
Choice of culture medium for the expansion of Th17 cells. (**a**) Cell growth after 3, 6, 8 or 13 days in RPMI (+FCS and 2 ng/mL TGF-β1) or IMDM (+10% KnockOut Serum Replacement) with different concentrations of recombinant human TGF-β1. (**b**) Proportions of IL-17A, IFN-γ and double positive cells after 6 days of culture as a function of TGF-β1 concentration (0 to 5 ng/mL). Results are means from the cells of two cows. (**c**) Proportions of IL-17A+ cells after 6 and 13 days of culture as a function of TGF-β1 concentration (0 to 5 ng/mL). Results are means from the cells of two cows. (**d**) Comparison of cell growth in RPMI and X-VIVO™ 15 media with or without polarizing cytokines (40 ng/mL IL-6 and 2 ng/mL TGF-β1), after 3 and 6 days of culture. (**e**) Proportions of cells IL-17A+ and IFN-γ+ (ICS) with or without polarizing cytokines (40 ng/mL IL-6 and 2 ng/mL TGF-β1) after 6 days of culture. Results are means from two cows.

We then decided to get rid of foetal calf serum, which is a variable source of cytokines and particularly TGF-β^29^ by replacing the RPMI + 10% FCS medium with a hematopoietic medium suitable for Th17 cell growth. This is of concern as TGF-β1 is considered an important driver of Th17 cell generation at low concentrations^30,31^. IMDM, a medium rich in aromatic amino acids, a source of ligands for the transcription factor AhR, has been advocated for the culture of mouse and human Th17 cells^32^. We cultured CD4+ T cells in IMDM + 10% KnockOut Serum Replacement under polarization condition, with various concentrations of TGF-β1 (0, 0.5, 2 or 5 ng/mL). The addition of TGF-β1 improved the growth of the CD4+ T cells, although cell growth was less in IMDM than with the RPMI medium (Fig. 4a). At day 6 of culture, the proportions of IL-17A+ cells in IMDM were about half of those in RPMI, independently of TGF-β1 concentration with a trend towards a reduction of double positive cells with increasing TGF-β1 concentration (Fig. 4b). However, proportions of IL-17A+ cells dropped dramatically at day 13, which suggests that the IMDM medium was not suitable for the growth of bovine Th17 cells under our conditions (Fig. 4c). The addition of TGF-β1 improved slightly the maintenance of IL-17A+ cells, independently of the tested concentration. On the basis of the growth-promoting effect of TGF-β1, we selected the concentration of 2 ng/mL for the subsequent experiments.

Then, we compared RPMI + FCS to the X-VIVO™ 15 medium with and without IL-6 (40 ng/mL) and TGF-β1 (2 ng/mL). The growth of CD4+ cells was comparable in the two media at day 3 without cytokines, but in favour of the X-VIVO™ 15 medium at day 6 in the presence of cytokines (Fig. 4d). Proportions of IL-17A+ cells and IL-17A-IFNγ double positive cells augmented in the presence of IL-6 + TGF-β1, and were similar or slightly higher in X-VIVO™ 15 than in RPMI + FCS (Fig. 4e). We also tested another medium (TexMACS™ medium, Miltenyi Biotec), which yielded results similar to the X-VIVO™ 15 medium (See Supplementary Fig. S1). According to these results, we settled on the use of the X-VIVO™ 15 medium and the addition of cytokines as described in the M&M section and Fig. 1.

### Surface labelling of IL-17A+ cells

**Figure 5:**
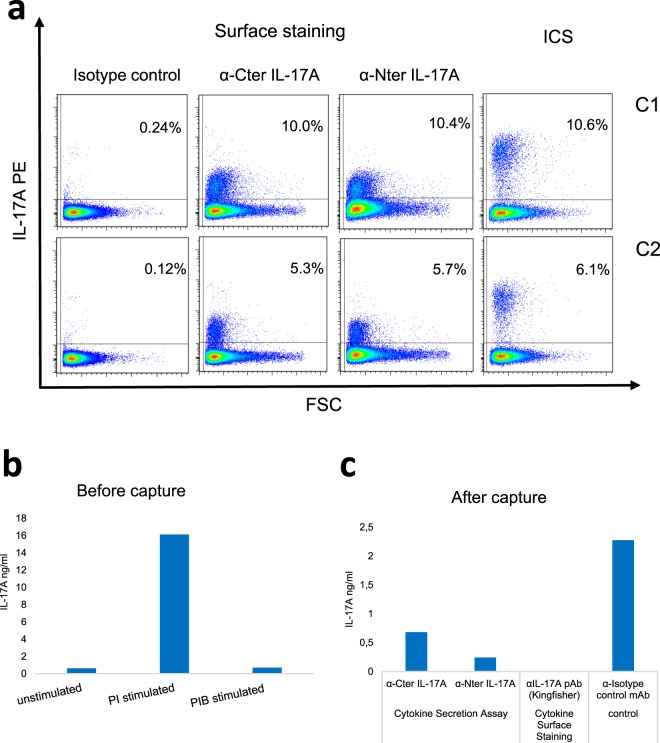
Efficiency of the monoclonal antibodies in the cytokine secretion assay. (**a**) Percentages of CD4+ T cells labelled by the capture complex with either the α-Cter or α-Nter monoclonals, and comparison with the percentages of IL-17A+ cells identified by ICS. Data from two cows (C1 & C2) are shown, one per row. (**b**) Concentrations of IL-17A in culture supernatant before capture, at the end of the 3.5 h of stimulation. (**c**) Concentrations of IL-17A in culture supernatant at the end of the secretion step (after capture) in the presence of the complex capture with either one of the two monoclonal antibodies to IL-17A, an isotype control or the polyclonal antiserum to IL-17A, showing the efficiency of the capture of IL-17A as it is secreted. PI: PMA/ionomycin, PIB: PMA/ionomycin/brefeldin A. ICS: intracellular staining.

Our first attempt to identify live IL-17A+ cells was by transposing to bovine cells the capture assay of secreted cytokine (CSA) developed for human cells^20^. We tested different combinations of antibodies that could allow us to capture and reveal the IL-17A secreted by stimulated CD4+ T cells (Supplementary Table S3). We began with commercially available antibodies that had proved to react with bovine IL-17A, but without success: the complex of streptavidin and biotinylated antibodies to CD45-IL-17A was revealed at the surface of the cells, but the detection antibodies did not enable IL-17A capture. Rabbit antibodies to bovine IL-17A (Kingfisher) were then used as capture antibody with monoclonal antibodies to bovine IL-17A N- or C-terminal amino acid sequences as detection antibodies. The stimulated lymphocytes were not labelled with these antibody combinations. Finally, we tested our monoclonal antibodies to bovine IL-17A as capture antibodies with the rabbit antibodies to bovine IL-17A (Kingfisher) followed by donkey anti-rabbit IgG-PE as detection antibodies. Both monoclonals gave similar positive results (Fig. 5a). Of note, the IL-17A capture complex recognized IL-17A-producing cells in similar quantities compared to intracellular staining (ICS) for IL-17A, as the proportion of IL-17+ cells detected using ICS and capture complex assay were comparable (Fig. 5a). A sizeable secretion of IL-17A occurred in culture supernatants at the end of the stimulation with PMA/ionomycin (PI), which was blocked by brefeldin A (PIB), as expected (Fig. 5b). We also measured IL-17A concentrations in culture supernatants after staining with the capture complex or directly with the polyclonal antibodies (CSA), at the end of the cytokine secretion step (Fig. 5c). Concentrations of IL-17A secreted in the medium (isotype control) were reduced by the capture complexes, by the anti-Nter slightly more efficiently than by the anti-Cter monoclonal antibody, and suppressed when the polyclonal antiserum (Kingfisher) was added. We then retained the combination anti-Nter monoclonal/anti-rabbit antibodies for the sorting of IL-17A-positive cells.

**Figure 6:**
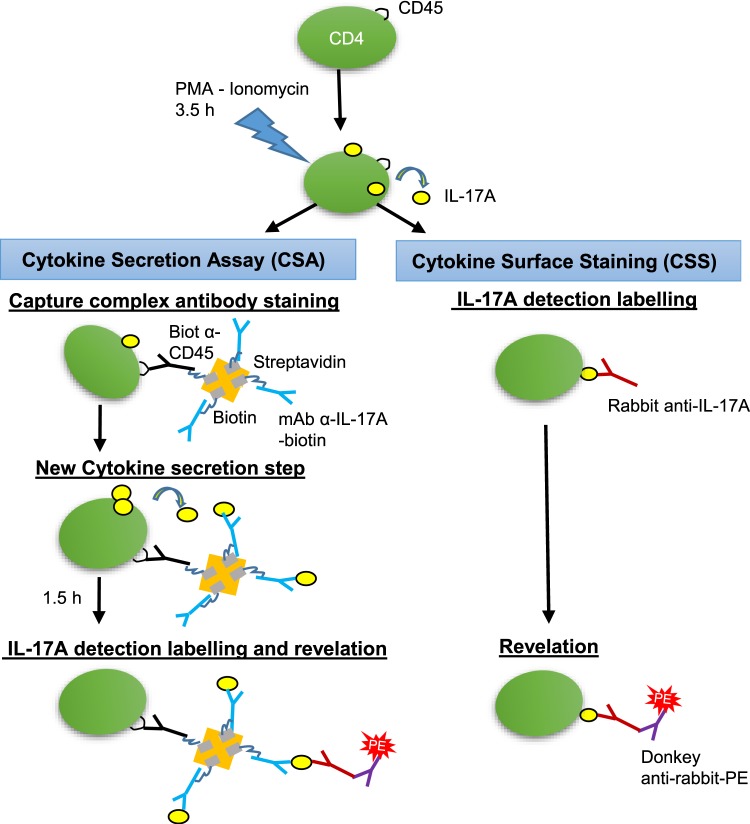
Schematic representation of the assays used for surface labelling of IL-17A secreting cells. In the cytokine secretion assay (CSA), after stimulation with PMA/ionomycin, the cells start to secrete cytokines for 3.5 h. Then the cells are allowed to bind the capture complex (Capture complex antibody staining) for 15 min before dilution and incubation for 1.5 h under agitation (New cytokine secretion step). The secreted cytokine is captured by antibodies to IL-17A that are maintained at the surface of the cell by antibodies to CD45. The capture complex comprises the two types of biotinylated antibodies linked by a streptavidin molecule. After washing, rabbit anti-bovine IL-17A antibodies are added that bind to the captured IL-17A (IL-17A detection labelling and revelation). Then the cells are washed and the binding of rabbit antibodies revealed with a secondary antibody conjugated to phycoerythrin. Alternatively, in the cytokine surface staining assay (CSS), after washing at the end of the 3.5 h stimulation, cells are incubated with rabbit antibodies to IL-17A, washed, and the binding of rabbit antibodies to surface-associated IL-17A is revealed with a secondary antibody conjugated to phycoerythrin.

**Figure 7:**
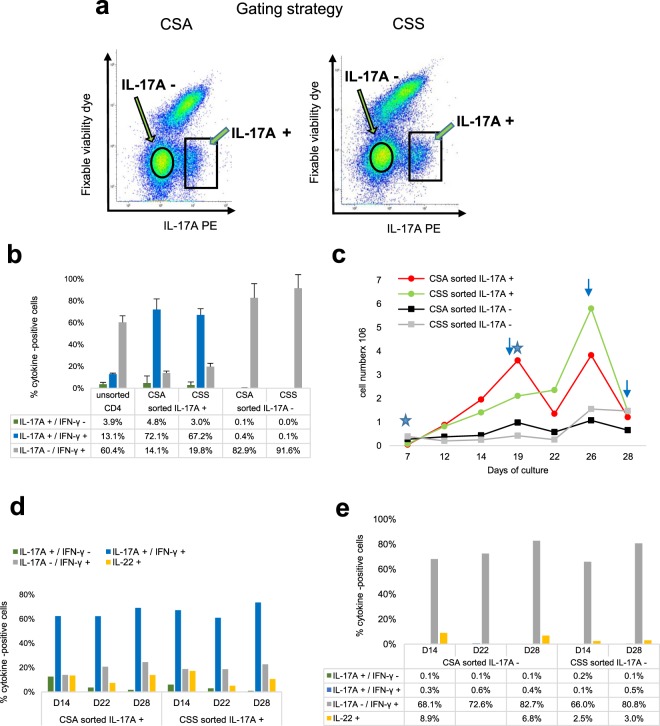
Comparison of the Cytokine Secretion Assay with the cytokine surface Staining. (**a**) Gating strategy. At the end of the culture expansion step, the polarized cell populations were split and analyzed side-by-side by flow cytometry with Cytokine Secretion (left) and surface staining (right) assays for IL-17A secretion. The squares indicate the IL-17A+ cells sorting windows, the circles the IL-17A- sorting windows. (**b**) Proportions of cytokine-positive cells 8 days after sorting. Median values from two sorting experiments with cells of two cows are shown. (**c**) Numbers of IL-17A+ and IL-17A- cells at different times after sorting. Stars indicate stimulations with anti-CD3/CD28 antibodies, arrows the sampling of cells for freezing or RNA preparation. Values are from one sorting experiment with two cows. (**d**) Proportions of sorted IL-17A+ cells producing (ICS) IL-17A, IFN-γ or IL-22 at different times after sorting. Values are from one sorting experiment with two cows. e) Proportions of sorted IL-17A- cells producing (ICS) IL-17A, IFN-γ or IL-22 at different times after sorting. CSA: cytokine secretion assay; CSS: cytokine surface staining.

A simpler technique of IL-17A+ cell surface labelling has been described for human cells^21^ (Fig. 6). This technique relies on the direct labelling of the IL-17A molecules that linger at the surface of secreting cells during the secretion window following stimulation with specific antibodies. We tested several of the antibodies used to develop the complex capture assay. The best results were obtained with the rabbit antibodies to bovine IL-17A (Kingfisher Biotech). The direct labelling of IL-17A+ CD4+ T cells with the rabbit antibodies was comparable to the labelling with the capture complex and yielded similar proportions of IL-17A+ cells (Fig. 7). Unstimulated cells and cells stimulated but treated with Brefeldin A were not labelled in the capture or direct assay of IL-17A surface labelling, which is in line with the capture of the cytokine as it is secreted and momentarily exposed at the surface of the cell.

### Sorting of Th17 cells, expansion and long-term culture of sorted Th17 cells

After stimulation (PMA/ionomycin) and labelling by using either the complex capture or the surface staining, IL-17A+ and IL-17A- cells were sorted by flow cytometry (Fig. 7a). From 10^6^ expanded CD4+ T cells, 3.5 to 9 × 10^4^ IL-17A+ cells were usually obtained. The IL-17A+ sorted cells were cultured in the polarizing X-VIVO™ 15 medium, the sorted IL-17A- cells in X-VIVO™ 15 medium without polarizing cytokines, both were stimulated once with anti-CD3 and anti-CD28 antibodies. On day 3 post-sorting and every third day, the medium was renewed and IL-2 added. The production of IL-17A and IFN-γ was tested by ICS when cells had grown to sufficient numbers, generally 7 to 8 days post-sorting. Usually, 70 to 80% of the sorted cells were IL-17A-positive by ICS 7 to 8 days post-sorting (D14-D15) (Fig. 7b). Most of IL-17A+ cells (about 90%) were also positive for IFN-γ, and the two sorting procedures yielded similar proportions of the two subsets (Fig. 7b). The sorted cells based on absence of surface IL-17A yielded mainly IFN-γ positive cells (80 to 90%) with very few IL-17A+ cells (less than 0.5%). Twelve to 14 days post-sorting, a new stimulation with anti-CD3 and anti-CD28 antibodies was carried out. Under these conditions, numbers of IL-17A+ cells increased steadily for at least 12 days and anew after another TCR stimulation (Fig. 7c). The IL-17A negative population could also be expanded, although it was consistently less proliferative (Fig. 7c). The proportions of IL-17A+ and IFN-γ+ cells remained rather constant on the culture period (21 days) in both sorted populations (Fig. 7d,e).

**Figure 8:**
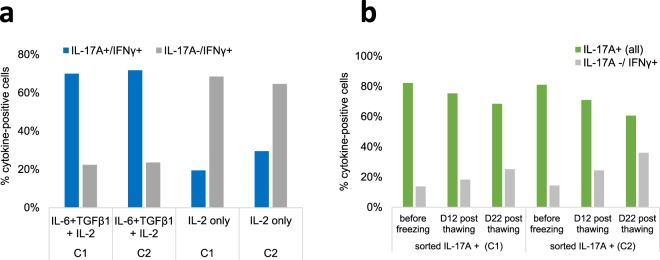
Maintenance of the IL-17A and IFN-γ phenotype of sorted and frozen-thawed cells upon subculture. (**a**) Sorted IL-17A+ cells stimulated with α-CD3/α-CD28 were cultured with IL-2 with or without the polarizing cytokines TGF-β1 and IL-6 for 9 days and analyzed by flow cytometry (ICS). Percentages of IL-17A+/IFN-γ+ double positive cells and of IL-17A-/IFN-g+ cells are shown. (**b**) Thawed IL-17A+ cells were cultured for 22 days and analyzed by ICS at days 12 and 22. Percentages of IL-17A+ cells (all: both IL-17A+/IFN-γ- and double positive cells) and IL-17A-/IFN-γ+ cells are shown. Results from two cows (C1 & C2) are shown.

We evaluated the capacity of the sorted IL-17A+ and IL-17A- CD4+ cells to withstand deep-frozen storage, thawing and subsequent maintenance in culture. Sorted Th17 cells were stimulated with antibodies to CD3/CD28 and cultured in X VIVO-15 medium with IL-2 or with the combination of IL-2 with the polarizing cytokines TGF-β1 and IL-6. It appeared that in the absence of the polarizing cytokines, most of the Th17 cells had become IL-17A negative at day 9 (Fig. 8a). We then evaluated the maintenance of the cytokine phenotype of frozen-thawed Th17 cells on prolonged culture. In the presence of the two polarizing cytokines, the percentages of IL-17A+ cells slightly decreased whereas the percentages of IL-17A− tended to increase (Fig. 8b).

### Cytokine production and transcriptomic profiling of the sorted Th17 cells

**Figure 9:**
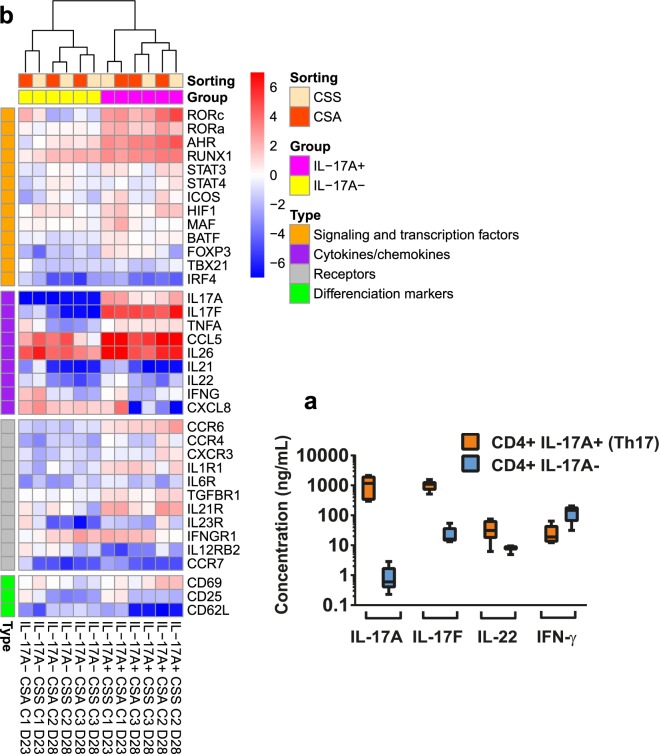
Unambiguous identification of bovine Th17. (**a**) Secretion of cytokines by sorted Th17 cells. After sorting, the cells were stimulated with α-CD3 and α-CD28 and cultured for 6 days. Supernatant contents were analysed by ELISA. Median values (10 to 90 percentiles) from five cell preparations from three cows are shown. (**b**) Comparison of Th17 signature gene expression between IL-17A+ and IL-17A- cells. Heatmap shows expression intensity expressed as log2(fold-change) normalized relative to three reference genes (ACTB, PPIA and GAPDH). Expression is relative to the CD4+ subset of the corresponding cow at day 6 after positive selection using MACS® beads. Cells were cultured in X-VIVO™ 15 medium without polarizing cytokines for 6 days after an initial TCR stimulation and a partial (half) renewal of medium with addition of IL-2 on the third day. Results are from three cows (C1, C2, C3).

The unambiguous identification of surface IL-17A+ cells as Th17 cells relies on the production of signature cytokines by the sorted populations. The secretion of cytokines by sorted IL-17A+ and IL-17A- cells was monitored by ELISA in culture supernatants after 6 days of culture (D14) (Fig. 9a). There was a tremendous difference in the production of IL-17A and IL-17F between the two cell types: IL-17A+ cells produced high concentrations, IL-17A- cells hardly produced IL-17A, and produced very little amounts of IL-17F (16.1 vs 1098 ng/mL for IL-17A+ cells). There was also some more production of IL-22 by IL-17A+ cells (31.2 ng/mL, median) than by IL-17A- cells (8.2 ng/mL, median), but more IFN-γ production by IL-17A- cells (147 vs 18.9 ng/mL, medians).

To address whether IL-17A production was associated with typical features of human or murine Th17 cells, a transcriptomic profiling was carried out to compare gene expression of IL-17A+ and IL-17A- cells at D23 or D28 (Fig. 9b). In accordance with ELISA results, there was a marked overexpression of IL-17A and IL-17F by IL-17A+ cells. Differences were less obvious for IL-22, IL-21 and IL-26, cytokines typically associated with Th17 cells. We also assessed the expression of genes encoding transcription factors associated with T helper cell subsets. As expected, the polarizing transcription factors *Rorc* and *Rora* were overexpressed by IL-17A+ cells, along with *AhR*. *Runx1* was expressed by both cell types. *Foxp3* was slightly expressed by IL-17A+ cells, whereas the expression of *Tbx21* appeared to be low, even in IL-17A- cells, possibly reflecting the low stimulation of these cells under our experimental conditions and despite sizeable IFN-γ production. The overexpression of CCR6 and IL21R are as expected from Th17 cells. It is worth bearing in mind that the results are expressed with reference to cells that had been TCR stimulated. Overall, the IL-17A+ cells displayed a characteristic Th17 gene signature. Of note, the Th17 cell preparations obtained by the two sorting procedures segregated together in the transcriptomic profile analysis (Fig. 9b), which adds to the similar quantitative results to support the interchangeability of the procedures.

## Discussion

In this study, we aimed at validating a procedure that enables the isolation of live bovine Th17 cells. To this end, we set out to enrich an initial cell population in IL-17A-producing cells. We started from blood CD4+ T cells, comprising both naïve and antigen-experienced cells, as shown by the 30% proportion of CD45R0+ cells. Enrichment was obtained by using TCR stimulation with antibodies to CD3 and CD28 in the presence of Th17-polarising cytokines. We used IL-6 and TGF-β1, a mixture that had proved efficient for human, murine and bovine IL-17A-producing cells^19,31,33^, even though some studies did not confirm the requirement for TGF-β to drive human Th17 differentiation^34,35^. That combination of cytokines has been shown to prompt naïve bovine CD4+ CD62L+ T cells to differentiate into Th17 cells^19^. At an early stage, we opted for a culture without foetal calf serum, which is a variable source of various cytokines and particularly TGF-β^29^. Among the different media and additives tested, the X-VIVO™ 15 medium proved to be appropriate for the expansion and maintenance of the T cells before and after isolation. As a function of the donor cow, between 5% and 15% of the expanded cells were IL-17A positive. These amounts were suitable for the development of a sorting procedure and its implementation.

Then, we sought to adapt to bovine cells a procedure initially developed for the isolation of murine Th2 cells^22^. This technique is based on the capture of secreted cytokines at the cell surface. This is achieved by using a bi-specific antibody complex binding both CD45 at the cell surface and the cytokine of interest as it is secreted. This approach has been used successfully to isolate human Th17 cells by flow cytometry cell sorting^20^. We selected antibodies to prepare the IL-17A-capture complex and the revelation fluorescence step. We obtained satisfactory results as judged by the similar proportions of IL-17A-positive cells yielded by the cell surface and intracellular labelling. The necessary reagents are commercially available for the isolation of human and murine IL-17A-producing cells (Miltenyi Biotec), but not for bovine cells. This is why we were interested in a simpler method based on the direct labelling of cell surface IL-17A that has been shown to be efficient for the isolation of human Th17 cells^21^. This approach proved to yield results similar to the cytokine secretion assay with bovine Th17 cells, with the advantage that all antibodies are commercially available. The cells obtained with either technique were cultivable and expandable. Their transcriptomic profile squared with the Th17 profile and they produced the Th17 signature cytokines IL-17A and IL-17F in high amounts.

Our culture conditions yielded workable proportions of IL-17A+ CD4+ cells, and were suitable for the multiplication of these cells. These conditions do not pretend to be the optimal and only culture conditions for bovine Th17 cells. There is no consensus over optimal conditions for the polarization of murine or human Th17 cells, as different conditions may generate Th17 cells with different phenotypes, with the confounding characteristic plasticity of these cells^30,31,35,36^. This topic constitutes a knowledge gap as far as bovine Th17 cells are concerned. We started from CD4+ T cells isolated from PBMC, thus including naïve and antigen-experienced cells. These cells produced high concentrations of IFN-γ under our culture conditions. IFN-γ has been shown to hinder the generation of Th17 cells, and anti-IFN-γ antibodies have been added to polarizing cocktails used to generate Th17 cells from naïve CD4+ T cells^1,34,37^. As commercialized antibodies to bovine IFN-γ are all sold with sodium azide and as neutralizing activity is not validated, we did not investigate the effect of anti-IFN-γ antibodies on the production of Th17 cells. The “refractory” status of our IL-17A+ cells to IFN-γ, their growth in the presence of IL-2, and the efficiency of anti-CD28 stimulations are compatible with the committed nature of the CD4+ that were expanded and stimulated under the conditions used in our study. It has been reported that under stimulation with anti-CD3/CD28, only CD45RO+ cells produce and secrete IL-17A, and that neutralizing IFN-γ and IL-4 does not affect IL-17 secretion by memory T cells (CD45RO+)^38^. Addition of IL-2 to the culture medium was necessary to expand the cells. It has been reported that IL-2 inhibits the early differentiation of Th17 cells but can drive the proliferation of differentiated Th17 cells^39,40^. Antibodies to CD28 have been shown to interfere with the development of human Th17 cells from naïve T cells^40^. Taking into account all these considerations, it is likely that the cells that expanded under our conditions were not naïve T cells. Thus, “polarizing conditions” might thus be an inappropriate designation for the conditions we used to obtain Th17 cells before sorting, as it could be mainly expansion rather than polarization. However, the loss of IL-17A production when IL-6 and TGF-β1 were omitted from the culture medium after sorting indicates that some polarizing drive was necessary to maintain the Th17 phenotype. This is in keeping with the requirement of TGF-β for sustained expression of IL-17A and IL-17F by murine Th17 cells^41^.To define the polarizing conditions for bovine Th17 cells, starting from naïve T cells will be necessary.

Another question that we did not answer is the need for IL-23 for the expansion or maintenance of bovine Th17 cells. Th17 cells express the receptor for IL-23. IL-23 is considered useful for survival and expansion of Th17 cells, even though some studies have shown that Th17 cells producing IL-17A (but not IFN-γ) can be generated without IL-23 and that TGF-β1 and IL-6 are sufficient for the Th17 differentiation *in vitro*^37,42^. The double production of IL-17 and IFN-γ by Th17 cells is favoured by IL-23 in mice^43^. IL-23 may also act on effector/memory Th17 cells to induce proliferation and to augment the production of IL-17A, IL17F and IL-22^44^. Under our conditions, bovine Th17 cells were mainly double producers of IL-17 and IFN-γ, even though the production of IL-17A and IL-17F exceeded largely the production of IFN-γ (Fig. 8a). In a preliminary experiment, we evaluated the effect of IL-23 on the multiplication of IL-17A+ cells on two occasions with two different recombinant human IL-23 cytokines, and did not observe noticeable modifications. In the absence of validation of activity of these proteins on bovine cells, we cannot reach a conclusion. Under our conditions, bovine Th17 produced rather low concentrations of IL-22. TGF-β has been reported to reduce the production of IL-22^31^. It is possible that our culture conditions did not favour the production of IL-22 by the purified Th17 cells, knowing that appropriate conditions remain to be established for bovine T cells.

In conclusion, we developed a set of procedures that makes it possible to expand, isolate and maintain bovine Th17 cells in culture. The two cell sorting procedures, using an IL-17A capture complex or a direct labelling of surface IL-17A, yielded similar quantitative and qualitative results. The direct labelling offers a rather simple isolation technique of viable and cultivable Th17 cells with commercial reagents. The isolated cells displayed the characteristics of Th17 cells, as assessed through the production of the signature cytokines IL-17A and IL-17F and the expression of the transcription factors genes *Rorc* and *Rora*. These techniques have the potential of enabling the isolation of Th17 and other IL-17A-producing cells and to investigate their activities in the bovine species.

## Supplementary information

Dataset 1

## Data Availability

The datasets generated during and/or analyzed during the current study are available from the corresponding author on reasonable request.
