# Supplementary material for: Expansion, isolation and first characterization of bovine Th17 lymphocytes
Source: Sci Rep. 2019 Nov 6;9:16115. doi: 10.1038/s41598-019-52562-2 (PMC6834651; doi:10.1038/s41598-019-52562-2)
Supplement: Supplementary file 1 — Dataset 1 [file 41598_2019_52562_MOESM1_ESM.pdf]

Expansion, isolation and first characterization of bovine Th17 lymphocytes

Patricia Cunha, Yves Le Vern, Christophe Gitton, Pierre Germon, Gilles Foucras & Pascal Rainard

Supplementary Tables

Supplementary Table S1. Antibodies used in the surface and intracellular phenotyping of bovine T lymphocytes

Antigen	Primary antibody	Host specificity	Clone	provider	dilution	Secondary antibody	provider	dilution
**Cell surface phenotyping**								
CD4	A647-conjugated mouse monoclonal	Bovine	CC8	Abd Serotec, Oxford UK	1/100			
CD45-RO	mouse monoclonal	Bovine	ILA116A	Kingfisher Biotech Saint Paul USA	1/200	PerCP conjugated goat anti-mouse IgG3	Interchim Jackson ImmunoResearch	1/200
CD62L	mouse monoclonal	Bovine	BAQ92A	Kingfisher Biotech Saint Paul USA	1/200	PE conjugated goat anti-mouse IgG1	Interchim Jackson ImmunoResearch	1/200
**Direct labelling of IL-17A**								
IL-17A	Rabbit polyclonal	Bovine		Kingfisher Biotech Saint Paul USA	1/200	RPE conjugated Donkey anti-rabbit IgG(H+L)	Interchim Jackson ImmunoResearch	1/200
**Intracellular phenotyping**								
IL-17A	PECy7-conjugated mouse monoclonal	Human	eBio64 DEC17	Thermo Fisher Scientific San Diego USA	1/25			
IFN-g	A488-conjugated mouse monoclonal	Bovine	CC302	Abd Serotec, Oxford UK	1/25			
IL-22	Rabbit polyclonal	Bovine		Produced In-house	1/200 5µg/ml	RPE conjugated Donkey anti-rabbit IgG(H+L)	Interchim Jackson ImmunoResearch	1/200

Supplementary Table S2. Antibody combinations used to develop the cytokine secretion assay (CSA)

Capture antibody(biotinylated)	Detection antibody & conjugate	Surface labelling
Rabbit α-bov-IL-17A (Kingfisher)	mAb α-hu-IL-17A-PE (eBio64DEC17)	Negative
Rabbit α-bov-IL-17A (Kingfisher)	Donkey α-rabbit IgG-PE	Complex α-CD45/IL-17 revealed
Rabbit α-bov-IL-17A (Kingfisher)	mAb α-bovIL-17A-Cter<br>Goat a-mouse IgG_1-PE	Negative
Rabbit α-bov-IL-17A (Kingfisher)	mAb α-bovIL-17A-Nter<br>Goat a-mouse IgG_1-PE	Negative
mAb α-bovIL-17A-Cter	mAb α-hu-IL-17A-PE (eBio64DEC17)	Negative
mAb α-bovIL-17A-Nter	mAb α-hu-IL-17A-PE (eBio64DEC17)	Negative
mAb α-bovIL-17A-Cter	Rabbit α-bov-IL-17A (Kingfisher)<br>Donkey α-rabbit IgG-PE	Positive +
mAb α-bovIL-17A-Nter	Rabbit α-bov-IL-17A (Kingfisher)<br>Donkey α-rabbit IgG-PE	Positive ++

Supplementary Table S3. ELISA procedure used to measure the production of Th17 signature cytokines

		IL-17A	IL-17F	IL-22
1-	Coating	Rabbit pAb anti-bovine IL-17A (Kingfisher Biotech)<br>PBS 0.5% gelatin<br>1 µg/ml	Rabbit pAb anti-bovine IL-17F (Immunogen : recombinant protein)<br>PBS 2% BSA<br>2µg/ml<br>100µl/p - Overnight 4°C	Rabbit pAb anti-bovine IL-22 (Immunogen : recombinant protein)<br>PBS 2% BSA+2% sucrose<br>1.5µg/ml
2-	Blocking (and dilution buffer)	PBS 0.5% gelatin<br>200µl/p – 1h 37°C	PBS 2% BSA<br>200µl/p – 1h RT	PBS 2% BSA<br>200µl/p – 1h 37°C
3-	Samples and standards	IL-17A transfected SF9 cells supernatant<br>1.5 à 10 ng/ml<br>100µl/p -2h 37°C	Bovine IL-17F recombinant protein (S2 cells production)<br>0.2 à 51 ng/ml<br>100µl/p -2h RT	Bovine IL-22 recombinant protein (S2 cells production)<br>0.05 à 3.2 ng/ml<br>100µl/p -2h 37°C
4-	Detection	Biotinylated Mouse mAb anti-IL-17A Nter peptide<br>0.5µg/ml<br>100µl/p -1h 37°C	Biotinylated Rabbit pAb anti-IL-17F (recombinant protein)<br>0.75µg/ml<br>100µl/p -1h RT	Biotinylated Rabbit pAb anti-IL-22 (recombinant protein)<br>1µg/ml<br>100µl/p -1h 37°C
5-	conjugate	Avidin HRP (Molecular Probes) 1/20 000 - 100µl/p<br>30 min 37°C	30 min RT	30 min 37°C
6-	Chromogen	100µl/p TMB (Uptima) – 15-30 min – Dark- RT		
7-	Stop solution	50µl/p HCL 1M		
8-	Read	DO 450 nm		

Supplementary Table S4. List of primers used for RT-qPCR

Gene symbol	Other name	Accession number	primer-5'	primer-3'	Amplicon (pb)
Signaling and transcription factors					
**RORc**	RORGt	NM_001083451	GGGCAGGGAGAACTTCTATG	TCTGCACCAGGTGCTCAATC	135
**RORa**		NM_001192861	GCAATGCCACCTACTCCTGT	GCGGCCAAATTTTACAGCATC	100
**TBX21**	Tbet	NM_001192140	CAGGGAACCGCCAGTATGTC	CACGATCATCTGGGTCGCAT	134
**FOXP3**		NM_001045933	GGTTTACACGCATGTTTGCC	TGCGGAACTCAAACTCATCC	147
**STAT3**		XM_024979867.1	GACCGGTGTCCAGTTCACAA	AAATTTCCGGGACCCTCTGA	133
**STAT4**		NM_001083692	ACAGCAAATCGCCTGCATTG	CTCCAACTGCCGTCTGAGTT	109
**AHR**		NM_001206026	GTGCAGAAAACTGTCAAGCCA	AACATCTGGTGGGAAAGGCAG	126
**ICOS**		NM_001034275	GCGTGCATGACCCTAACAGT	GGTGCCAGGGAGTTCCAAAT	104
**HIF1**		NM_174339	ACCCTGCACTCAACCAAGAA	TGGGACTGTTAGGCTCAGGT	150
**MAF**	VMAF	NM_001105637	CCGTCCTCTCCCGAGTTTTT	CCGGGTGGGTCTGAGAGAAT	115
**IRF4**		NM_001206162	AGCCTGTGAAAATGGTTGCC	TCGGCAGACCTTATGCTTGG	126
**BATF**		NM_001206278	CGGCAAACAGGACTCATCTG	GCGTTCTGCTTCTCCAAGTC	144
**RUNX1**		NM_001256578	GGGTGCCTTTTCAGGAGGAA	CTGGCATCGTGAACGTCTCT	127
Differenciation markers					
**CD25**	IL2RA	NM_174358	AACACACAGATGCGCAGAAC	TTCACGTTCGTGTTCCCATG	95
**CD69**		NM_174014	AACAGAGACCAGCTCCTTGC	GCCCACTGATAGAGCAACGA	169
**CD62L**	selectin L	NM_174182	ATGCCTGCCACAAAGCAAAG	TTCCACACATTGTCCATGGC	86
Receptors					
**IL-23R**		NM_001127172	GATCGCTTCGAACTGGGATT	GGACCTGTTCGCTGGAATTG	150
**IL-21R**		NM_001193179	TGCGACTTTGCTCCTGCTAA	ATGTGTCATGCCAGGTGAGG	146
**TGFBR1**		NM_174621	TACCCAAGGAAAACCAGCCA	TTGTATCCGTGGCCGAATCA	128
**IL-1R1**	CD121a	NM_001206735	TCCTTCTCTGGAGGCTGATAA	GGCGTCGTGCTGTCATTTTT	150
**IL-12RB2**		NM_174645	ATCTGTGTGGGGCAGAGTTG	TCCACCACGTATTCCTGCAC	127
**IL-6R**		NM_001110785	TCAGTGCCGTGGACAGAAAC	CGGATCCTTCAGCATCAGTGT	149
**IFN-GR1**		NM_001035063	ACCCAAATCCTTGCTGTCTG	TGTGCAGGCTTGAAATGGTC	140
**CCR4**		NM_001100293	GAAAAGCAAGGGCCTGGAAAAA	AAGAACCTGGAACGCACCT	103
**CCR6**		NM_001194961	AGAAAGCCAAGTCCATGACG	TGGCGTTGCTGAAAATCCAC	126
**CCR7**		NM_001024930.3	AGCAAGCAACTCAACATCGC	AGGCGTACAAGAAAGGGTTG	82
**CXCR3**		NM_001011673	CCACAGGACTTCAGCCTCAA	CGACTGCCACGATGCCATTA	100
Produced Cytokines and chemokines					
**IL-17A**		NM_001008412	GCCCACCTACTGAGGACAAG	GCTGGATGGTGACAGAGTTC	246
**IL-17F**		XM_582420	CACTCTGGAGGACCACATTG	GAGTTCAGGGTCCTGTCTTC	216
**IL-21**		NM_198832	GTGGCCCATAAGTCAAGCTC	CGCTCACAGTGTCTCTTTAC	152
**IL-22**		NM_001098379	AGGAGCCCTACATCTTCAAC	CTTCGTCACCTGATGGATTC	122
**IL-26**		XM_001250651	CAGAGCAACGATTCCAGAAG	TCTGCCTGAGGCTATGAAAG	194
**IFN-G**		NM_174086	ACCAGGTCATTCAAAGGAGCAT	TCTGCAGATCATCCACCGGA	100
**TNF-A**		NM_173966.3	TCTTCTCAAGCCTCAAGTAACAAGC	CCATGAGGGCATTGGCATAC	104
**CCL5**		NM_175827	CTGCCTTCGCTGTCCTCCTGATG	TTCTCTGGGTTGGCGCACACCTG	217
**IL-8**	CXCL8	NM_173925.2	TGAAGCTGCAGTTCTGTCAAG	TTCTGCACCCACTTTTCCTTGG	202
Reference genes					
**ACT B**		BT030480	ACGGGCAGGTCATCACCATC	AGCACCGTGTTGGCGTAGAG	166
**GAPDH**		DQ403066	GGCATCGTGGAGGGACTTATG	GCCAGTGAGCTTCCCGTTGAG	187
**PPIA**		BC105173	TCCGGGATTTATGTGCCAGGG	GCTTGCCATCCAACCACTCAG	206

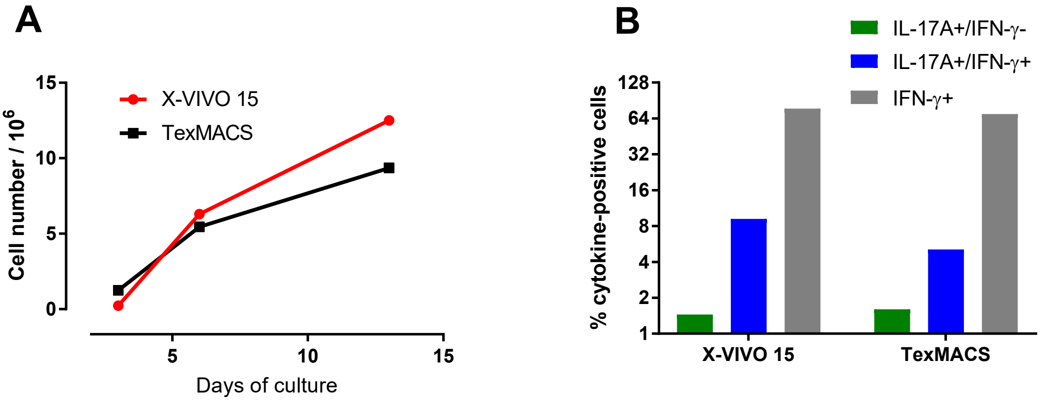

Supplementary Figure S1. Comparison of cell growth in X-VIVO 15 and TexMACS media supplemented with polarizing cytokines (TGF-β1 and IL-6) and with addition of IL-2 at days 3 and 6. A) Number of cells after 3, 6 and 13 days of culture. B) Proportions of cytokine-positive cells (IL-17A or IFN-?) at day 14.
